# The Effect of Pramipexole Therapy on Balance Disorder and Fall Risk in Parkinson's Disease at Early Stage: Clinical and Posturographic Assessment

**DOI:** 10.5402/2012/320607

**Published:** 2012-08-05

**Authors:** Sibel Güler, Levent Sinan Bir, Beyza Akdag, Fusun Ardıc

**Affiliations:** ^1^Department of Neurology, Faculty of Medicine, Trakya University, 22030 Edirne, Turkey; ^2^Department of Neurology, Faculty of Medicine, Pamukkale University, 20070 Denizli, Turkey; ^3^Department of Biostatistics, Faculty of Medicine, Pamukkale University, 20070 Denizli, Turkey; ^4^Department of Physical Treatment and Rehabilitation, Faculty of Medicine, Pamukkale University, 20070 Denizli, Turkey

## Abstract

The aim of this study was to determine balance problems and severity and ratio of postural instability of newly diagnosed, early stage Parkinson's patients who did not receive any antiparkinson treatment before, to evaluate fall risk clinically and posturographically and to examine the effects of pramipexole on these signs and symptoms. Detailed posturographic assessments which involved central vestibular, visual, peripheric vestibular somatosensory field tests were applied to both patient and control subjects and fall risk was determined. There was not statistically significant difference between patients and control subjects before and after drug therapy in the assesment of fall risk in posturography and there was not any improvement with drug usage in the patient group. However, in the analysis of subsystems separately, only the involvement in central vestibular field was more severe and could appear at all positions in Parkinson's patients comparing with the control group, and pramipexole was partially effective in improving this disorder. Central vestibular field is the subsystem that should be examined with first priority. Posturography is relatively reliable in defining fall risk and postural instability ratio in Parkinson's disease. But it should be considered that clinical assessment tools can be more sensitive in the evaluation of balance and postural disorders and in the follow-up of the response to drug therapy.

## 1. Introduction

Parkinson's Disease (PD) is a common neurodegenerative disease which affects 1-2% of the population over 60 years old [1]. It was defined by James Parkinson in the year 1817 as shaking palsy, the most common type of parkinsonism (bradykinesia, resting tremor, rigidity, impairment in postural reflexes, flexion posture, and freezing phenomenon) [2]. Balance disorder (BD) is the least specific symptom of PD; however, it is the cardinal symptom, which is the main cause of disability and it definitively affects the function ability of the patients in their daily living activities [3]. It is found in 96% of all Parkinson's disease patients in long-term followups [4]. Fall risk ratio is reported to be 38% in Parkinson's patients [5]. Pramipexole (PM) is an aminothiazole derivative, a nonergo dopamine agonist, which is selective for D2 dopaminergic receptors and also effective for D3 receptors [6, 7]. Posturography is the method of measuring the balance, which is a combined test protocol developed to enable systematical documentation of balance disorders. It was used in this study for a more detailed and objective assessment of the balance functions, to find out which subsystem/systems were the source of the problem, and to monitorize the effect of drug. There are few articles in which BD in early stages of PD were examined. The aim of our study was to define ratio and severity of BD at early stages and to assess the effect of a dopamine antagonist on this finding clinically and posturographically, which had been used commonly at early stage. 

## 2. Materials and Methods

### 2.1. Patients and the Evaluated Parameters

19 male, 12 female, total 31 early-middle stage newly diagnosed PD patients and 31 voluntary subjects who were matched from sex and age point of view and not having any neurological disease were included into the study. The patients were selected from subjects who applied to University Hospital, Outpatient Clinic of Movement Disorders between December 2007 and August 2008 with the confirmation of University Hospital Ethics Committee, dated November, 26 2007 and numbered 11. All the participants were informed about the aim and scope of the study and their informative consents were taken. The patients who did not receive L-Dopa or any dopaminergic treatment before, did not have an indication for starting L-DOPA treatment, and did not have any indication for L-DOPA were included into the study. Detailed histories of the subjects were taken and their physical and neurological examinations were done. PM therapy was started to the patients, planning to increase the dose up to 3 mg within six weeks gradually. The tests were applied 3 times to patient group and 2 times to control group, in order to define the changes that could occur as a result of learning. The parameters which were used and recorded during the statistical analysis were age, sex, PD duration, family history, and Hoehn&Yahr (H&Y) and Unified Parkinson's Disease Rating Scale (UPDRS) scores of subjects obtained without any drug usage, with 1.5 mg PM and with 3 mg PM; the three different drug doses score at Berg Balance Scale (BBS) in patient group, the scores of first test and the test repeated after 1.5 months in control group and the global fall risk percent found at posturography with 3 different PM doses in patient group and with 2 tests reported in control group; the number of subjects with deprivation in any of the test positions in central vestibular, visual, peripheric vestibular, and somatosensorial fields; the number of positions that any deprivation was found in any of four fields; the sum of weight ratio of deprivations in all positions in four fields, respectively. 

### 2.2. Clinical Measurements

H&Y scale, used for classification of stages in PD, was applied to the patients. The disease was classified as 1–5. Also UPDRS used for defining the severity of disease (mental state, daily living activities, and motor performance) was applied to patients. Any difference at scores with drug therapy was assessed. Both patient and control groups were assessed with BBS, a clinical balance test which is highly sensitive and reliable, which consists of 14 different activities. 

### 2.3. Posturography

**Figure 1:**
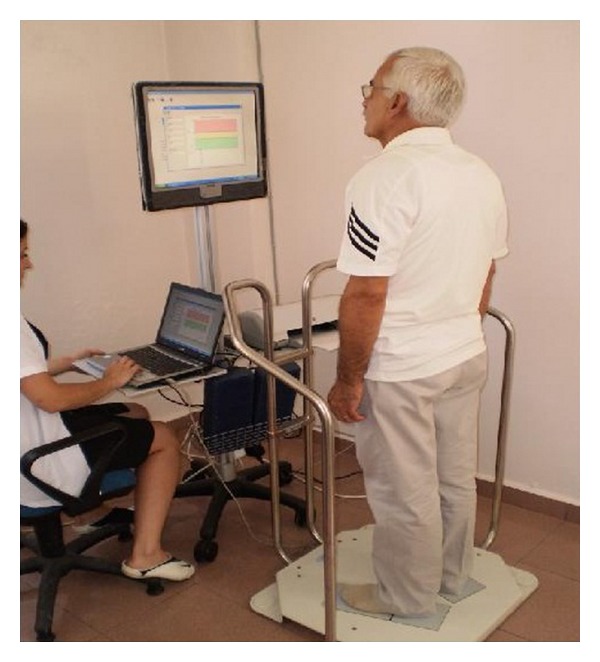
Balance measurement with posturography.

**Figure 2:**
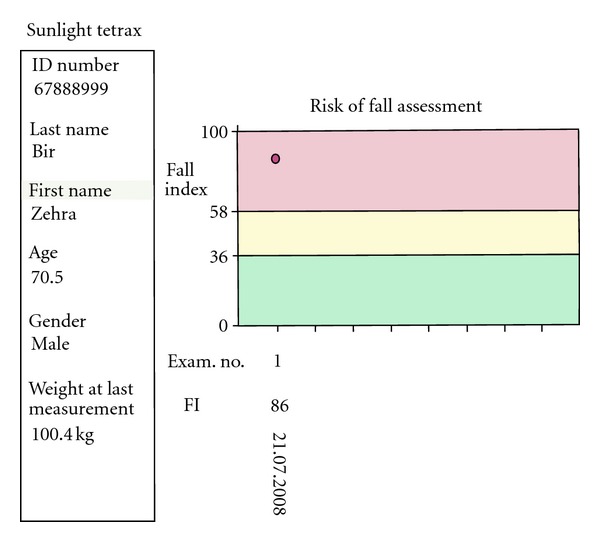
Assessment at fall risk.

**Figure 3:**
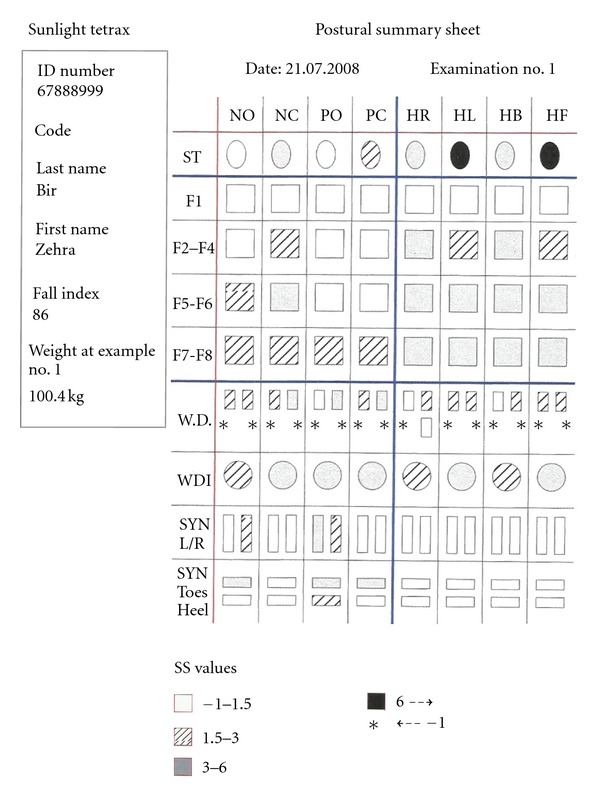
Posture summary report.

**Figure 4:**
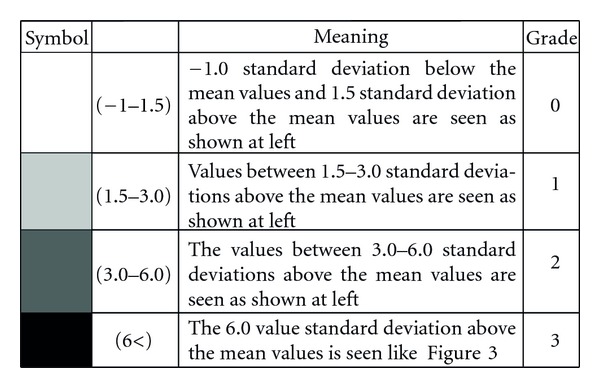
Grading scale.

Measurements of both patient and control groups were done using Tetrax (Sunlight Medical Ltd Israel) posturography device, whose main principle is to monitor postural oscillation and can evaluate the balance objectively. The subjects were taken into the test room and were taken on the platform with pressure sensors with their shoes off (Figure 1). Tests lasted totally 4 minutes with each of them lasting 32 seconds at eight positions that included standing in straight gait with eyes open and closed (limits visual input), standing on soft plate with eyes open and closed (limits somatosensorial input), and head rotated right and left with eyes closed and head flexion, extension with eyes closed (Table 1), and the pressure changes of the leg were measured. The global fall risk index scores of patients and control groups obtained from eight different positions were examined by posturographic measurement, dividing the results into 3 categories (Figure 2). The subjects with fall risk in green area (0–36%) were evaluated to have low-level fall risk, while subjects with fall risk in yellow area (37–58%) were evaluated to have middle-level fall risk and the subjects with fall risk in pink area (59–100%) were evaluated to have high fall risk. The fall risk percent was used as an absolute parametric value in statistical analysis. After the general measurement in which we assessed global fall risk of the patients and control groups in eight positions, posture summary report based on standing oscillations intensity in different frequencies was examined and evaluated, in order to to define how far the central vestibular, visual, peripheric vestibular, and somatosensory fields were affected and to clarify which field was more effective in causing global fall risk (Figure 3). The symbols in posture summary report are consisted of shadows at different tones or black boxes. Shadow grade is dyed in accordance with the deviation of the patient performance from normal values. Darker shadows mean a positive deviation. Lighter shadows mean getting closer to normal values. After defining the number of subjects in which a deprivation was found at any test positions in central vestibular, visual, peripheric vestibular, somatosensory field, the number of positions in which impairment was found in the subjects with deprivation at any test position was analysed. At last stage, the sum of weight ratio of impairments in all positions was calculated in every subject. Since this result could not be given as an absolute value on posturography, grading method was used (Figure 4).

### 2.4. Statistics

Student's *t*-test and Pearson Chi-Square tests were used to compare age values and sex, Friedman's two-way analysis of variance, Wilcoxon with Bonferroni correction matched two samples test, and Marginal homogenity tests were used for clinical scores, H&Y, UPDRS, and BBS scores and posturography results analysis in patients group. Mann Whitney *U* test and Chi-Square test were used for the comparison of BBS scores and posturography results between patient and control groups. BBS scores and posturography results were compared with Wilcoxon 2 related samples test and Chi-Square test in control group. The threshold of significance was accepted as 0.05 for all tests. 

## 3. Results

### 3.1. Clinical Results (Table 2)

The mean age of patients group was 71.8 ± 8.60 (56–90) and the mean age of the control group was found 71.54 ± 6.98 (58–88) years. 61.3% (*n* = 19) of the patients group was male, 38.7% (*n* = 12) of them was female. 51.6% (*n* = 16) of the control group was male and 48.4% (*n* = 15) was female. The differences of average age and sex between two groups were not significant (Student *t* test *P* > 0.05, Pearson Chi-Square test *P* = 0.609, resp.). The statistically significant difference as a decrease between H&Y stage scores before drug usage, 1.5 mg and 3 mg PM, in patients group (*P* < 0.001, Friedman's two-way analysis of variance) was also found in dependent samples (*P* < 0.001, Wilcoxon 2 related samples test with Bonferroni correction). The difference as a decrease between UPDRS scores in 3 different measurements was significant (*P* < 0.001, Friedman's two-way analysis of variance), while this difference also persisted in dependent groups (*P* < 0.001, Wilcoxon 2 related samples test with Bonferroni correction). In the evaluation by BBS, the significant difference as an increase between patients scores without drug usage and control scores also persisted (*P* < 0.001, Mann-Whitney *U* test) between patients with 3 mg PM usage and control group measurements (*P* < 0.001, Mann-Whitney *U* test) done 1.5 months later. Also statistically significant difference as an increase in 3 BBS scores of patient group (*P* < 0.001, Friedman's two-way analysis of variance), continued in dependent groups (*P* < 0.001, Wilcoxon 2 related samples test with Bonferroni correction). There was not any significant difference between two BBS tests in the control group (*P* < 0.001, Wilcoxon 2 related samples test) (All clinical measurement results are summarized in Table 3). 

### 3.2. Posturographic Results

There was not any significant difference in global fall risk percents between the patients without drug usage and the first measurements of the control group also in global fall risk percents between patients using 3 mg PM drug therapy and measurements after 1.5 month of the control group (Mann-Whitney *U* test, *P* = 0.442, *P* = 0.712, resp.). 

There was not any significant difference in the analysis of the drug effect on global fall risk percent in the measurements of the patients group before drug usage, 1.5 mg PM, and 3 mg PM usage (*P* < 0.05, Friedman's two-way analysis of variance).

The number of subjects in the patient group in whom an impairment was found in any position in central vestibular field without drug usage was 27 (87%) while this number in the control group was 25 (80.6%) for the first test. Impairment was shown in 24 subjects (77.4%) among the patients group using 3 mg PM. 23 (74.19%) participants among the control group developed impairment in any of test positions of this field in the assessment after 1.5 months. There was not a statistically significant difference in comparison between patient-control groups (*P* > 0.05, Pearson's Chi-Square test).

The number of patients in whom an impairment was found in any positions in central vestibular field was 27 (87%) before drug usage, 27 (87%) with 1.5 mg PM, and 24 (77.4%) with 3 mg PM. There was not any statistically significant difference between these 3 measurements in which the effects of the drug on patient numbers were compared (*P* > 0.05, Marginal homogenity test). 

There was not any statistically significant difference in the comparison of position numbers of the patient group without drug usage in which any impairment was found in central vestibular field and the first test of the control group, and also there was not a significant difference in comparisons between patients with 3 mg PM usage and second test of the control group (*P* = 0.542, *P* = 0.293, Mann-Whitney *U* test). 

 The difference in the decrease found in the comparison of results of 3 tests to assess the effect of drug on the number of positions in which any impairment was found in central vestibular field in the patient group was statistically significant (*P* < 0.001, Friedman's two-way analysis of variance). This statistically significant difference found to persist when all the dependent groups were compared (*P* = 0.010, Wilcoxon 2 related samples test with Bonferroni correction).

 The difference as an increase in the sum of weight ratios of the impairments in all positions in the patients group was significant when compared with the first test of the control group (*P* = 0.001, Mann-Whitney *U* test). The difference between the patients of whom the effect of the drug was evaluated when using 3 mg PM and the control during the second comparison was found to be nonsignificant (*P* > 0.05, Mann-Whitney *U* test).

The difference between the values obtained during 3 measurements in which the effect of the drug on the sum of weights ratio of the impairments obtained in all positions in the central vestibular field in the patients was evaluated, was found to be statistically significant (*P* < 0.001, Friedman's two-way analysis of variance). This difference between all groups found to persist when the dependent groups were compared again (*P* < 0.001, Wilcoxon 2 related samples test with Bonferroni correction). (Posturography results are summarized in Table 3).

## 4. Discussion

Falling is a situation that can result in severe complications, even death in geriatric patients. Posture and balance disorders are not taken into consideration and even can be ignored at early stages of PD. Therefore, the drugs developed for symptomatic treatment are rather aimed to treat tremor and bradykinesia [8]. 

This study focused on postural stability impairment, balance disorder and fall risk which are cardinal symptoms observed in PD and have an important contribution on disability and decrease in quality of life [3]. There are not sufficient and detailed studies about how severe and common this problem, seen quite clear and in important percentage in advanced stages, occurs at early stage. Therefore, we aimed to find out what the posture and balance status was at the early stages and the effects of a commonly used treatment regimen. Nonergo agonists became the first choice in symptomatic treatment because ergot derivative dopamine agonists found to have side effects like valvulopathy and fibrosis. This is the basic reason why we used PM in our study. Other reasons were being easy-to-use and the fact that dose standardization can be achieved in shorter time more similarly [9]. Advanced age necessarily causes many problems that may affect posture and balance. Such problems include firstly degenerative changes in visual, peripheric vestibular and in locomotor systems, and even cardiovascular diseases and drugs. Unfortunately, the same problems affect the age-matched control group. It is almost impossible to constitute a simple healthy group with only parkinsonian symptoms without any effect in all the other subsystems and a control group in the same age. Therefore, our results should be interpreted carefully. 

There is not a specific scale dealing with the assessment of PI. PI is usually assessed by the number of falls during the last 6 months [10]. Visser et al. suggested that the most valid test is pushing back test (pushing back from shoulders without a notice) in the assessment of balance in PD [11]. In our study, we observed postural imbalance in 20 patients (64.5%) in their examination of postural balance, in which the patients went back but could recover without help thereafter, though there was not a full postural unresponsiveness, and was considered a mild degree postural imbalance. As a result, it was seen in our study that PI ratio assessed with easy push back test which relied on balance adjustment speed was 2/3. When PI severity was not as much as postural unresponsiveness and was assessed over 4 points in easy-pushing back test, 20 cases (64.5%) were found to be grade 1. Consequently, it was observed that the patients at early stage could be in balance without support by the help of compensatory mechanisms and there were postural responses in many of the subjects, though there was postural imbalance. 

First of all, we applied clinical assessment scales to our patients. It is shown in the literature that PM improved H&Y scale and decreased the “off” duration in L-DOPA treatment [12]. In parallel to the literature, we also showed in our study that PM improved H&Y scale in parallel to dose increase. 

 Qutubuddin et al. reported that PM improved UPDRS scores significantly and that this improvement was also shown during the measurements after 2, 4, 8, and 12 weeks [13]. Similarly, we observed in our study a decrease in UPDRS scores during the measurements before and after drug usage in correlation with the clinical improvement. 

There are only very few studies examining which test in Parkinson's patients measures the balance best [14–19]. Shumway-Cook et al. found the average BBS scores as 52.6 in elderly without fall history and as 39.6 in elderly with fall history and they indicated that this test was an important predictor of falls in the elderly [20]. The highest score (41–56 points) [21, 22] in BBS shows the best balance. Our study showed that the statistically significant difference between BBS scores of patients and control groups before the patients starting their PM therapy persisted in the second measurement of the patients when having 3 mg PM. However, it was observed that the significant difference found in BBS scores during the measurements before and after drug usage, in which PM effect was assessed in patient group, persisted between the dependent groups. This result was important since it showed the effect of PM on balance problem in PD. 

The fall risk index of the subject is calculated with the help of the pressure center and gravity center oscillation angles through posturography [8]. There was no significant difference in comparisons between patients and control groups when the patients did not use any drug and when using 3 mg PM. There was no significant difference in comparisons at 3 measurements in the patients group. The interpretation of this finding was that PM was not effective on global fall risk, or objective methods like posturography were not sensitive as much as general analysis in which all the systems were evaluated.

In posturography, the analyses of the subsystems are highly sensitively performed [23]. There was no statistically significant difference in the comparisons of the number of subjects who had any impairment in any test positions in each of four systems between patient and control groups before and after drug use. Thus, PM was not sufficiently effective to cause a total decrease in the number of patients with balance disorder and fall risk.

A secondary subtest done by posturography, namely the analyses in which the number of positions with any impairment was found, was repeated in four subsystems. A statistically significant difference was only found in the central vestibular system, showing that the number of positions with any impairment in the measurements before drug usage, with 1.5 mg PM and 3 mg PM, decreased with PM therapy in patient group in which the effect of drug use was examined. As our device does not give an absolute value for the severity of disorder, the sum of weight ratio of impairments in all positions was examined again by using grading method. In this test, which was our third subtest, there was a statistically significant difference only in the first test between the patients and the control group only in central vestibular field. It was observed that this difference disappeared in the second test which was performed while the patients were having 3 mg PM. We defined that there was statistically significant difference in this test which was repeated 3 times in patients group and this difference persisted also in the dependent subgroups. As a result, PM was found to be effective on the central vestibular system. 

Clinical BBS assessments showed that there was a significant difference between Parkinson's patients and the control group, which was compatible with manifest balance disorder when compared with the control group. The positive effect of PM on balance was determined clinically. It was seen on posturographic evaluation that the factors, which cause a similar global fall risk in patient group and raised up the fall risk by affecting the age-matched control group, contained the clinical balance scales like BBS less. This situation showed that clinical assessment was more sensitive than paraclinical tests in monitorization of the fall risk and treatment in PD. It was shown that there was a more severe impairment in Parkinson's patients than in control group in central vestibular field when analysing subsystems separately in posturography.

In conclusion, in this study it is defined that PI, which usually is ruled out at the early stages of PD, can be found in a grade that does not cause a loss in postural control in Parkinson patients. It is shown that the main cause of PI and balance disorder is the impairment in central vestibular field and this symptom can be treated partially with PM therapy. 

## Figures and Tables

**Table 1:** Posturography tests: test positions (NO-HF).

	Standing position	Head position	Eye position
NO	Without pads	Head erect	Eyes open
NC	Without pads	Head erect	Eyes closed
PO	On pillows	Head erect	Eyes open
PC	On pillows	Head erect	Eyes closed
HR	Without pads	Head rotated 45 degrees to the right	Eyes closed
HL	Without pads	Head rotated 45 degrees to the left	Eyes closed
HB	Without pads	Head backwards	Eyes closed
HF	Without pads	Head forward	Eyes closed

**Table 2:** Clinical results data.

	Patients group	Control group	Patient group	Control group	Patient group	
	(before drug usage)	(baseline)	(1.5 mg PM)	(After 1.5 months)	(3 mg PM)	*P*
	Mean	Mean	Mean	Mean	Mean	
H&Y (mean)	2		1.5		1	<0.001
UPDRS (sum-mean)	33		28		22	<0.001
UPDRS (mental-mean)	3		2.5		2	<0.001
UPDRS (DLA-mean)	13.5		12.5		9	<0.001
UPDRS (M-mean)	13.5		12.5		8.5	<0.001
BBS (PvC-mean)	51	54				<0.001
BBS (PvC-mean)				55	53	<0.001
BBS (P-mean)	51		52		53	<0.001

DLA: daily living activities, P: patient, C: control, and M: motor.

**Table 3:** Statistical data of posturographic examination of patient and control groups.

	Patients group	Control (baseline)	Patient	Patient	Control	
	(before drug usage)	(first test)	(1.5 mg PM)	(3 mg PM)	(after 1.5 months)	*P*
			(1.5 mg PM)	(after 1.5 month)	(3 mg PM)	
	Mean	Mean	Mean	Mean	Mean	
GFRP (PvC-mean)	56.30	52.96				>0.05
GFRP (PvC-mean)				54.07	49.74	>0.05
GFRP (P-mean)	56.30		50.38	54.07		>0.05
GFRP (C-mean)		52.96			49.74	>0.05
NSDCVFP (PvC)	27	23				>0.05
NSDCVFP (PvC)				24	25	>0.05
NSDCVFP (P)	27		27	25		>0.05
NSDCVFP (C)		23			25	>0.05
NBDCVFP (PvC-mean)	4.23	4.00				>0.05
NBDCVFP (PvC-mean)				3.65	4.2	>0.05
NBDCVFP (P-mean)	4.23		3.38	3.65		<0.001
NBDCVFP (C-mean)		4.00			4.2	>0.05
SWRDP (PvC-mean)	2.12	0.67				<0.001
SWRDP (PvC-mean)				0.26	0.51	>0.05
SWRDP (P-mean)	2.12		0.77	0.26		<0.001
SWRDP (C-mean)		0.67			0.51	>0.05

GFRP: global fall risk in posturography, NSDCVFP: number of subjects that found impaired in any test positions in central vestibular field on posturography, NBDCVFP: the number of positions in which any impairment was found in the central vestibular field on posturography, SWRDP: sum of weight ratio of defects in all positions in central vestibular field on posturogaphy. P: patient, C: control.
